# The top 50 most-cited articles about COVID-19 and the complications of COVID-19: A bibliometric analysis

**DOI:** 10.12688/f1000research.145713.1

**Published:** 2024-02-16

**Authors:** Tanya Singh, Jagadish Rao Padubidri, Pavanchand H. Shetty, Matthew Antony Manoj, Therese Mary, Bhanu Thejaswi Pallempati

**Affiliations:** 1 Kasturba Medical College Mangalore, Manipal Academy of Higher Education, Manipal, India; 2 Department of Forensic Medicine and Toxicology, Kasturba Medical College Mangalore, Manipal Academy of Higher Education, Manipal, India

**Keywords:** COVID-19, Complications, Bibliometry Analysis, Citations, SARS-COV-2

## Abstract

**Background:**

This bibliometric analysis examines the top 50 most-cited articles on COVID-19 complications, offering insights into the multifaceted impact of the virus. Since its emergence in Wuhan in December 2019, COVID-19 has evolved into a global health crisis, with over 770 million confirmed cases and 6.9 million deaths as of September 2023. Initially recognized as a respiratory illness causing pneumonia and ARDS, its diverse complications extend to cardiovascular, gastrointestinal, renal, hematological, neurological, endocrinological, ophthalmological, hepatobiliary, and dermatological systems.

**Methods:**

Identifying the top 50 articles from a pool of 5940 in Scopus, the analysis spans November 2019 to July 2021, employing terms related to COVID-19 and complications. Rigorous review criteria excluded non-relevant studies, basic science research, and animal models. The authors independently reviewed articles, considering factors like title, citations, publication year, journal, impact factor, authors, study details, and patient demographics.

**Results:**

The focus is primarily on 2020 publications (96%), with all articles being open access. Leading journals include The Lancet, NEJM, and JAMA, with prominent contributions from Internal Medicine (46.9%) and Pulmonary Medicine (14.5%). China played a major role (34.9%), followed by France and Belgium. Clinical features were the primary study topic (68%), often utilizing retrospective designs (24%). Among 22,477 patients analyzed, 54.8% were male, with the most common age group being 26-65 years (63.2%). Complications of COVID-19 affected 13.9% of patients, with a recovery rate of 57.8%.

**Conclusion:**

Analyzing these top-cited articles offers clinicians and researchers a comprehensive, timely understanding of influential COVID-19 literature. This approach uncovers attributes contributing to high citations and provides authors with valuable insights for crafting impactful research. As a strategic tool, this analysis facilitates staying updated and making meaningful contributions to the dynamic field of COVID-19 research.

## Introduction

In December 2019, the first outbreak of Coronavirus disease (COVID-19) was detected in Wuhan. ^1^ On 30 ^th^ January 2020, the World Health Organization (WHO) declared COVID-19 outbreak as a “public health emergency of international concern” and on 11 ^th^ March 2020, COVID-19 was declared a pandemic by the WHO. ^2^ As of September 2023, the coronavirus SARS-COV-2 (severe acute respiratory syndrome coronavirus-2) is responsible for a total of 770 million confirmed cases and 6.9 million confirmed deaths. With the start of COVID-19 vaccination, a total of 3 billion vaccine doses have been administered as per WHO. ^3^ While, SARS-COV-2 is primarily considered as a respiratory disease known to cause pneumonia and acute respiratory distress syndrome (ARDS), there have been numerous reports about its many extra pulmonary manifestations. Literature suggests that the cardiovascular, gastrointestinal, renal, haematological, neurologic, endocrinologic, ophthalmologic, hepatobiliary and dermatologic systems can all be affected. ^4^ This can result either due to the extrapulmonary dissemination and replication of the SARS-COV-2 or due to the widespread immunopathological sequelae of the disease. ^5^

Bibliometric analysis is the application of statistical methods to evaluate the impact of a manuscript, research performance, author contribution to a particular field and to quantitatively analyse the most influential articles related to a particular field. ^6^

According to our literature search, no current bibliometric analysis has focused on COVID-19 complications. The purpose of this bibliometric analysis was to compile and analyse the top 50 most-cited articles regarding COVID-19 complications across all peer-reviewed scientific journals.

## Methods

A bibliometric analysis of the most cited articles about COVID-19 complications was conducted in July 2021 using all journals indexed in Elsevier’s Scopus and Thomas Reuter’s Web of Science from November 1, 2019 to July 1, 2021. All journals were selected for inclusion regardless of country of origin, language, medical speciality, or electronic availability of articles or abstracts. The terms were combined as follows:

(“COVID-19” OR “COVID19” OR “SARS-COV-2” OR “SARSCOV2” OR “SARS 2” OR “Novel coronavirus” OR “2019-nCov” OR “Coronavirus”)

AND

(“Complication” OR “Long Term Complication” OR “Post-Intensive Care Syndrome” OR “Venous Thromboembolism” OR “Acute Kidney Injury” OR “Acute Liver Injury” OR “Post COVID-19 Syndrome” OR “Acute Cardiac Injury” OR “Cardiac Arrest” OR “Stroke” OR “Embolism” OR “Septic Shock” OR “Disseminated Intravascular Coagulation” OR “Secondary Infection” OR “Blood Clots” OR “Cytokine Release Syndrome” OR “Paediatric Inflammatory Multisystem Syndrome” OR “Vaccine Induced Thrombosis with Thrombocytopenia Syndrome” OR “Aspergillosis” OR “Mucormycosis” OR “Autoimmune Thrombocytopenia Anaemia” OR “Immune Thrombocytopenia” OR “Subacute Thyroiditis” OR “Acute Respiratory Failure” OR “Acute Respiratory Distress Syndrome” OR “Pneumonia” OR “Subcutaneous Emphysema” OR “Pneumothorax” OR “Pneumomediastinum” OR “Encephalopathy” OR “Pancreatitis” OR “Chronic Fatigue” OR “Rhabdomyolysis” OR “Neurologic Complication” OR “Cardiovascular Complications” OR “Psychiatric Complication” OR “Respiratory Complication” OR “Cardiac Complication” OR “Vascular Complication” OR “Renal Complication” OR “Gastrointestinal Complication” OR “Haematological Complication” OR “Hepatobiliary Complication” OR “Musculoskeletal Complication” OR “Genitourinary Complication” OR “Otorhinolaryngology Complication” OR “Dermatological Complication” OR “Paediatric Complication” OR “Geriatric Complication” OR “Pregnancy Complication”) in the Title, Abstract or Keyword.

A total of 5940 articles were accessed, of which the top 50 most cited articles about COVID-19 and Complications of COVID-19 were selected through Scopus. Each article was reviewed for its appropriateness for inclusion. The articles were independently reviewed by three researchers (JRP, MAM and TS) ( Table 1). Differences in opinion with regards to article inclusion was resolved by consensus.

**Table 1.** Top 50 most cited articles about COVID-19 and the complications of COVID-19.

Rank	Article	Number of citations	Average number of citations per year
1.	Huang C, Wang Y, Li X, Ren L, Zhao J, Hu Y *et al.* Clinical features of patients infected with 2019 novel coronavirus in Wuhan, China. Lancet 2020; 395 (10223): 497-506	24775	8258
2.	Zhou F, Yu T, Du R, Fan G, Liu Y, Liu Z, *et al.* Clinical course and risk factors for mortality of adult inpatients with COVID-19 in Wuhan, China: a retrospective cohort study. Lancet 2020; 395(100229): 1054-1062	14341	4780
3.	Wang D, Hu B, Hu C, Zhu F, Liu X, Zhang J *et al.* Clinical Characteristics of 138 Hospitalized Patients With 2019 Novel Coronavirus–Infected Pneumonia in Wuhan, China. *JAMA* 2020; 323(11): 1061–1069	12740	4247
4.	Chen N, Zhou M, Dong X, Qu J, Gong F, Han Y, *et al.* Epidemiological and clinical characteristics of 99 cases of 2019 novel coronavirus pneumonia in Wuhan, Chine: a description study. Lancet 2020; 395 (10223): 507-513	11163	3721
5.	Zhou P, Yang XL, Wang XG, Hu B, Zhang L, Zhang W, *et al.* A pneumonia outbreak associated with a new coronavirus of probable bat origin. Nature 2020; 579: 270-273	10780	3593
6.	Wu F, Zhao S, Yu B, Chen MY, Wang W, Song GZ, *et al.* A new coronavirus associated with human respiratory disease in China. Nature 2020; 579: 265-269	5417	1806
7.	Yang X, Yu Y, Xu J, Shu H, Xia J, Liu H, *et al.* Clinical course and outcomes of critically ill patients with SARS-COV-2 pneumonia in Wuhan, China: a single centered, retrospective, observational study. Lancet Respir Med 2020; 8(5): 475-481	5369	1790
8.	Mehta P, McAuley DF, Brown M, Sanchez E, Tattersall RS, Manson JJ. COVID-19: consider cytokine storm syndromes and immunosuppression. Lancet 2020; 395(10229): 1033-1034	5196	1732
9.	Richardson S, Hirsch JS, Narasimhan M, Crawford JM, McGinn T, Davidson KW *et al.* Presenting Characteristics, Comorbidities, and Outcomes Among 5700 Patients Hospitalized With COVID-19 in the New York City Area. JAMA 2020; 323(20): 2052-2059	4811	1604
10.	Wu C, Chen X, Cai Y, Xia J, Zhou X, Xu S *et al.* Risk Factors Associated with Acute Respiratory Distress Syndrome and Death in Patients with Coronavirus Disease 2019 Pneumonia in Wuhan, China. JAMA Intern Med 2020; 180(7): 934-943	4427	1476
11.	Wang C, Pan R, Wan X, Tan Y, Xu L, Ho CS, *et al.* Immediate Psychological Responses and Associated Factors during the Initial Stage of the 2019 Coronavirus Disease (COVID-19) Epidemic among the General Population in China. Int J Environ Res Public Health 2020; 17(5): 1729	4371	1457
12.	Baden LR, Sahly HM, Essink B, Kotloff K, Frey S, Novak R, *et al.* Efficacy and safety of the mRNA-1273 SARS-CoV-2 vaccine. N Engl J Med 2021; 384: 403-416	3758	1879
13.	Mao L, Jin H, Wang M, Hu Y, Chen S, He Q, *et al.* Neurologic Manifestations of Hospitalized Patients With Coronavirus Disease 2019 in Wuhan, China. JAMA Neurol 2020; 77(6): 683-690	3576	1192
14.	Cao B, Wang Y, Wen D, Liu W, Wang J, Fan G, *et al.* A trial of lopinavir-ritonavir in adults hospitalized with severe covid-19. N Engl J Med 2020; 382: 1787-1799	3228	1076
15.	Cucinotta D, Vanelli M. WHO Declares COVID-19 a Pandemic. Acta Biomed 2020; 91(1):157-60	2774	925
16.	Ruan Q, Yang K, Wang W, Jiang L, Song J. Clinical predictors of mortality due to COVID-19 based on analysis of data of 150 patients from Wuhan, China. Intensive Care Med 2020; 46: 846-848	2633	878
17.	Holmes EA, Connor RC,Perry VH, Tracey I, Wessely S, Arseneault L, *et al.* Multidisciplinary research priorities for the COVID-19 pandemic: a call for action for mental health science. The Lancet Psychiatry 2020; 7(6): 547-560.	2628	876
18.	Liang W, Guan W, Chen R, Wang W, Li J, Xu K, *et al.* Cancer patients in SARS-COV-2 infection: a nationwide analysis in China. Lancet Oncol 2020; 21(3): 335-337	2600	867
19.	Klok FA, Kruip MJHA, Van der Meer NJM, Arbous MS, Gommers DAMPJ, Kant KM, *et al.* Incidence of thrombotic complications in critically ill ICU patients with COVID-19. Thromb Res 2020; 191: 145-147	2479	826
20.	Chen G, Wu D, Guo W, Cao Y, Huang D, Wang H *et al.* Clinical and immunological features of severe and moderate coronavirus disease 2019. J Clin Invest 2020; 130(5): 2620-2629	2473	824
21.	Shi S, Qin M, Shen B. Association of cardiac injury with mortality in hospitalised patients with COVID-19 in Wuhan, China. JAMA Cardiol 2020; 5(7): 802-810	2347	782
22.	Guo T, Fan Y, Chen M, *et al.* Cardiovascular implications of fatal outcomes of patients with coronavirus disease 2019 (COVID-19). JAMA Cardiol 2020; 5(7): 811-818	2225	742
23.	Chen T, Wu D, Chen H, Yan W, Yang D, Chen G *et al.* Clinical characteristics of 113 deceased patients with coronavirus disease 2019: retrospective study. BMJ 2020; 368: m1091	2187	729
24.	Chen H, Guo J, Wang C, Luo F, Yu X, Zhang W *et al.* Clinical characteristics and intrauterine vertical transmission potential of COVID-19 infection in nine pregnant women: a retrospective review of medical records. Lancet 2020; 395(10226): 809-815	2172	724
25.	Shi H, Han X, Jiang N, Cao Y, Alwalid O, Gu J, *et al.* Radiological findings from 81 patients with COVID-19 pneumonia in Wuhan, China: a descriptive study. The Lancet Infectious Diseases, Volume 20, Issue 4, 425-434	2124	708
26.	Zhang JJ, Dong X, Cao YY, Yuan YD, Yang YB, Yan YQ, *et al.* Clinical characteristics of 140 patients infected with SARS-COV-2 in Wuhan, China. Allergy 2020; 75(7): 1730-1741	2084	695
27.	Wang Y, Zhang D, Du G, Du R, Zhou J, Jin Y *et al.* Remdesivir in adults with severe COVID-19: a randomised, double-blind, placebo-controlled, multicentre trial. The Lancet 2020; 395(10236): 1569-1578	2016	672
28.	Tang N, Bai H, Chen X,Gong J, Li D, Sun Z. Anticoagulant treatment is associated with decreased mortality in severe coronavirus disease 2019 patients with coagulopathy. J Thromb Haemost 2020; 18: 1094-1099	1997	666
29.	Hui DS, Azhar EI, Madani TA, Ntoumi F, Kock R, Dar O, *et al.* The continuing 2019-nCov epidemic threat of novel coronaviruses to global health- The latest 2019 novel coronavirus outbreak in Wuhan, China. Int J Infect Dis 2020; 91: 264-266	1859	620
30.	Fang Y, Zhang H, Xie J, Minjie L, Ying L, Pang P, *et al.* Sensitivity of Chest CT for COVID-19: Comparison to RT-PCR. Radiology 2020; 296(2): E115-E117	1859	620
31.	Peiris JSM, Chu CM, Cheng VCC, Chan KS, Hung IFN, Poon LLM *et al.* Clinical progression and viral load in a community outbreak of coronavirus-associated SARS pneumonia: a prospective study. Lancet 2003; 361(9371): 1767-1772	1793	90
32.	Zheng YY, Ma YT, Zhang JY, *et al.* COVID-19 and the cardiovascular system. Nat Rev Cardiol 2020; 17: 259–260	1785	595
33.	Grein J,Ohmagari N, Shin D, Diaz G, *et al.* Compassionate use of Remdesivir for patients with severe covid-19. N Engl J Med 2020; 382: 2327-2336	1692	564
34.	Carfi A, Bernabei R, Landi F. Persistent symptoms in patients after acute COVID-19. JAMA 2020; 324(6): 603-605	1588	529
35.	Chung M, Bernheim A, Mei X, Zhang N, Huang M, Zeno X. CT imaging features of 2019 novel coronavirus (2019-nCoV). Radiology 2020; 295(1): 202-207	1587	529
36.	Helms J, Tacquard C, Severac F, *et al.* High risk of thrombosis in patients with severe SARS-CoV-2 infection: a multicenter prospective cohort study. Intensive Care Med 2020; 46:1089-1098	1531	510
37.	Bhatraju PK, Ghassemieh BJ, Nichols M, Kim R, Jerome KR, Naila AK, *et al.* Covid-19 in critically ill patients in the Seattle region-Case series. N Engl J Med 2020; 382: 2012-2022	1505	502
38.	Lechien JR, Chinese-Estomba CM, De Siati DR, *et al.* Olfactory and gustatory dysfunctions as a clinical presentation of mild-to-moderate forms of the coronavirus disease (COVID-19): a multicenter European study. Eur Arch Otorhinolaryngol 2020; 277: 2251-2261	1448	483
39.	Zhang H, Penninger JM, Li y, *et al.* Angiotensin-converting enzyme 2 (ACE2) as a SARS-CoV-2 receptor: molecular mechanisms and potential therapeutic target. Intensive care Med 2020; 46: 586-590	1443	481
40.	Cheng Y, Lou R, Wang K, Yao Y, Ge S, Xu G, *et al.* Kidney disease is associated with in-hospital death of patients with COVID-19. Kidney Int 2020;97(5): 829-838	1425	475
41.	Helms J, Kramer S, Merdji H, Clere-Jehl R, Schenck M, Kummerlen C, *et al.* Neurologic features in severe SARS-CoV-2 infection. N Engl J Med 2020; 382: 2268-2270	1415	472
42.	Wichmann D, Sperhake JP, Lutgehetmann M, Steurer S, Edler C, Heinemann A, *et al.* Autopsy findings and venous thromboembolism in patients with COVID-19. Ann Intern Med 2020; 173 (4): 268-277	1351	450
43.	Reynolds HR, Adhikari S, Pulgarin C, Troxel AB, Iturrate E, Johnson SB, *et al.* Renin-angiotensin-aldosterone system inhibitors and risk of covid-19. N Engl J Med 2020; 382: 2441-2448	1338	446
44.	Russell CD, Millar JE,Baillie JK. Clinical evidence does not support corticosteroid treatment for 2019-nCoV lung injury. Lancet 2020; 395(10223): 473-475	1332	444
45.	Kanji M, Katsushi K, Alexander Z, Gerardo C. Estimating the asymptomatic proportion of coronavirus disease 2019 (COVID-19) cases on board the Diamond Princess cruise ship, Yokohama, Japan, 2020. Euro Surveill 2020; 25 (10): 2000180	1330	443
46.	Arentz M, Yim E, Klaff L, Lokhandwala S, Riedo FX, Chong M. Characteristics and outcomes of 21 critically ill patients with COVID-19 in Washington state. JAMA 2020; 323(16): 1612-1614	1317	439
47.	Oxley TJ, Mocco J, Majidi S, Kellner CP, Shoirah H, Singh IP, *et al.* Large-vessel stroke as a presenting feature of Covid-19 in the young. N Engl J Med 2020; 382: e60	1314	438
48.	Macro C, Mulvey JJ, Berlin D, Harp J, Baxter-Stoltzfus A, Laurence J. Complement associated microvascular injury and thrombosis in the pathogenesis of severe COVID-19 infection: A report of five cases. Transl Res 2020; 220: 1-13	1294	431
49.	Goyal P, Choi JJ, Pinheiro LC, Schenck EJ, Chen R, Jabri A, Stalin MJ, *et al.* Clinical characteristics of Covid-19 in New York City. N Engl J Med 2020; 382: 2372-2374	1271	424
50.	Monteil V, Kwon H, Prado P, Hagelkruys A, Wimmer RA, Stahl M. Inhibition of SARS-CoV-2 infections in engineered human tissues using clinical-grade soluble human ACE2. Cell 2020; 181(4): P905-913	1251	417

The inclusion criteria specified articles that were focused on COVID-19 and Complications of COVID-19. Articles were excluded if they did not relate to COVID-19 and or complications of COVID-19, Basic Science Research and studies using animal models or phantoms. Review articles, Viewpoints, Guidelines, Perspectives and Meta-analysis were also excluded from the top 50 most-cited articles ( Table 1).

The top 50 most-cited articles were compiled in a single database and the relevant data was extracted. The database included: Article Title, Scopus Citations, Year of Publication, Journal, Journal Impact Factor, Authors, Number of Authors, Department Affiliation, Number of Institutions, Country of Origin, Study Topic, Study Design, Sample Size, Open Access, Non-Original Articles, Patient/Participants Age, Gender, Symptoms, Signs, Co-morbidities, Complications, Imaging Modalities Used and outcome.

## Results

### Year of publication

Of the total 50 articles that were analyzed, 48 (96%) articles were published in the year 2020, while 1 (2%) article was published in 2021 and 1 (2%) article was published in the year 2003 ( Table 2).

**Table 2.** Year of publications of the top 50 most-cited COVID-19 articles.

Year of Publication	N (50)	%
2020	48	96
2021	1	2
2003	1	2

### Open access

All of the 50 (100%) articles analyzed in the bibliometric study were open access articles.

### Journals

Of the total 50 articles that were analyzed, the journals that published the most number of articles published were in the The Lancet (9), NEJM (7) and JAMA (4), while Eurosurveillance, Transitional Research and Annals of Internal Medicine published one each ( Table 3).

**Table 3.** Peer-reviewed journals that published the top 50 most cited articles of COVID-19.

Journal	N (50)	%
The Lancet	9	18
NEJM	7	14
JAMA	4	8
Springer link	3	6
nature	2	4
JAMA cardiology	2	4
Radiology (RSNA)	2	4
Others	21	42

### Authors

Among the 50 articles that were analyzed, the most frequently cited authors contributed 4 articles each to the list ( Table 4).

**Table 4.** Authors contributing to the top 50 most-cited COVID-19 articles.

Name of the author	N
Yeming Wang	4
Zhang Li	4
Guohui Fan	4
Jiuyang Xu	4
Xiaoying Gu	4
Ting Yu	4
Yuan Wei	4
Bin Cao	4

### Departmental affiliations

Among the top 50 most-cited articles, the departmental affiliations were primarily in Internal Medicine (46.9%), Pulmonary Medicine (14.5%), and Bioscience and Technology (12.1%). Conversely, the Departments of Immunology, Paediatrics, and Urology had the lowest representation ( Table 5).

**Table 5.** Departmental affiliations of the authors contributing to the top 50 most-cited COVID-19 articles.

Department	N (595)	%
Internal Medicine	279	46.9
Pulmonary Medicine	86	14.5
Bioscience and Biotechnology	72	12.1
Critical Care and Anaesthesiology	32	5.4
Psychiatry	27	4.5
Laboratory Medicine	24	4.0
CTVS	21	3.5
Radiology	18	3.0
Public health and statistics	14	2.4
OBG	10	1.7
Others	12	2.0

### Country of origin

In the research analyzed within the top 50 COVID-19 articles, a total of 83 countries participated. Notably, China played a substantial role, contributing to 29 articles (34.9%). France and Belgium closely followed; each being involved in 10 articles (12.1%). Additional contributing nations included Canada with 3 articles (3.6%), the UK with 2 articles (2.4%), and Switzerland with 1 article (1.2%) ( Table 6).

**Table 6.** Countries involved in the top 50 most-cited COVID-19 articles.

Country	N (83)	%
China	29	34.9
France	10	12.1
Belgium	10	12.1
USA	9	10.8
Italy	5	6.0
Spain	4	4.8
Canada	3	3.6
Others	13	15.7

### Study topic

Among the 50 most-cited articles examined in this analysis, the predominant research themes were clinical features, comprising 34 articles (68%). Following closely were articles related to drug trials and interactions, accounting for 6 articles (12%). Additionally, there were 3 articles (6%) dedicated to analytical studies. Mortality studies constituted 2 articles (4%), while a single article each (2%) delved into the psychological impact and molecular mechanisms of COVID-19 ( Table 7).

**Table 7.** Study topic distribution among the top 50 most-cited articles.

Study topic	N (50)	%
Clinical Features	34	68
Drug Trials and Interactions	6	12
Analysis and Surveillance	3	6
Laboratory Investigations	2	4
Mortality Study	2	4
Others	3	6

### Study design

Among the 50 most-cited articles examined, the most common study designs were retrospective (24%), followed by prospective (16%) and correspondence (12%). Other study designs included prospective cohort (4%), randomized controlled trial (4%), and cross-sectional (2%) ( Table 8).

**Table 8.** Study designs among the top 50 most-cited COVID-19 articles.

Study design	N (50)	%
Retrospective	12	24
Prospective	8	16
Correspondence	6	12
Case Series	5	10
Comment	4	8
Retrospective Cohort	3	6
Prospective Cohort	2	4
Randomised Controlled Trial	2	4
Others	8	16

### Patient statistics/demographics

A total of 22477 patients were analyzed from the top 50 most-cited articles.

### Gender

Of the total 22477 patients, 12318 (54.8%) were males while 10159 (45.2%) were females ( Table 9).

**Table 9.** Gender distribution among the patients with COVID-19 from the top 50 most-cited COVID-19 articles.

Gender	N (22477)	%
Male	12318	54.8
Female	10159	45.2

### Age distribution

The most common age group among the patients was 26-65 years of age with 14255 (63.2%) patients, followed by the age of 65 years with 6018 (26.8%) patients and then 12-25 years with 2204 (9.8%) patients ( Table 10).

**Table 10.** Age distribution among the patients with COVID-19.

Age	N (22477)	%
12-25 years	2204	9.8
26-65 years	14255	63.4
>65 years	6018	26.8

### Clinical features

Among the 22477 patients, the most common clinical features were fever in 6333 (28.2%) patients, tachycardia in 4022 (17.9%) patients and dry cough in 3107 (13.8%) patients. Other common features included impaired sense of smell in 412 (1.8%) patients, impaired taste sensation in 404 (1.8%) patients and headache in 330 (1.5%) patients ( Table 11).

**Table 11.** Clinical features among the patients with COVID-19 from the top 50 most-cited articles.

Clinical features	N (22477)	%
Fever	6333	28.2
Tachycardia	4022	17.9
Dry Cough	3107	13.8
Tachypnoea	2113	9.4
Fatigue	1812	8.1
Myalgia	1785	7.9
Dyspnoea	1337	5.9
Productive Cough	514	2.3
Diarrhoea	500	2.2
Nausea	449	1.9
Impaired smell	412	1.8
Impaired taste	404	1.8
Others	2023	9.0

### Investigations

A total of 22477 patients underwent RT-PCR testing and were positive for COVID-19. Out of which, 18055 (80.3) patients underwent additional laboratory investigations. CT-chest was done for 11110 (49.4%) patients, chest X-ray was done for 8327 (22.7%) patients, and ECG done for 5098 (22.7%) patients. MRI brain and EEG were done for 8 (0.04%) patients with neurological complications ( Table 12).

**Table 12.** Investigations used in patients with COVID-19.

Investigations	N (22477)	%
RT-PCR	22477	100
Lab investigations	18055	80.3
CT chest	11110	49.4
X-ray chest	8327	37.1
ECG	5098	22.7
MRI brain, EEG	8	0.04

### Comorbidities

Out of the total 22477 patients, 21989 (97.8%) patients were found to have comorbidities. Of the 21989 patients with comorbidities, hypertension was present in 6720 (30.6%) patients, diabetes mellitus in 5082 (23.1%) patients and obesity in 2666 (12.1%) patients. Other comorbidities found included chronic liver disease in 74 (0.34%) patients, endocrine disorders in 53 (0.24%) patients and psychiatric disorders in 13 (0.06%) patients ( Table 13).

**Table 13.** List of comorbidities among the patients analyzed from the top 50 most-cited articles.

Comorbidities	N (21989)	%
Hypertension	6720	30.6
Diabetes mellitus	5082	23.1
Obesity	2666	12.1
Chronic Kidney Disease	1605	7.3
Chronic Obstructive Pulmonary Disease	1357	6.2
Congestive Heart Failure	1091	4.9
Coronary Heart Disease	782	3.6
Cardiovascular Disease	721	3.3
Asthma	576	2.6
Cancer	435	1.9
Others	954	4.3

### Complications

Out of the total 22477 patients, 3143 (13.9%) patients developed complications. The most common complication seen among the patients were acute kidney injury in 707 (22.5%) patients, ARDS in 576 (18.3%) patients and Sepsis in 347 (11.0%) patients. Other complications included electrolyte imbalance in 81 (2.6%) patients, acute hepatic injury in 74 (2.4%) patients and arrhythmias in 63 (2.0%) patients ( Table 14).

**Table 14.** Complications among the patients analyzed from the top 50 most-cited articles.

Complications	N (3143)	%
Acute Kidney Injury	707	22.5
Acute Respiratory Distress Syndrome	576	18.3
Sepsis	347	11.0
Respiratory Failure	295	9.4
Lower Respiratory Tract Infection	194	6.2
Myocardial injury	184	5.9
Shock	183	5.8
Heart Failure	119	3.8
Pulmonary Embolism/Deep Vein Thrombosis	115	3.6
Electrolyte imbalance	81	2.6
Others	342	10.9

### Outcome

Of the total 22477 patients who tested positive with COVID-19, 12981 (57.8%) recovered without any complications, while 6998 (31.1%) patients were hospitalised, and 2498 (11.1%) patients deceased due to the illness ( Table 15).

**Table 15.** Outcomes of the patients with COVID-19.

Outcome	N (22477)	%
Recovered/Discharged	12981	57.8
Hospitalised	6998	31.1
Dead	2498	11.1

## Discussion

In the bibliometric analysis of the top 50 most-cited COVID-19 articles, the most cited article was “Clinical features of patients infected with 2019 novel coronavirus in Wuhan, China” by Huang C et al., in The Lancet published in 2020. This article has the highest total number of citations (24775) as well as the highest average number of citations per year (8258) among the top 50 most-cited articles.

The Lancet published the greatest number of articles from the top 50 most-cited list with a total of 9 articles, followed by NEJM with 7 articles and JAMA with 4 articles published respectively. Most of the articles (n=48, 96%) were published in the year 2020 with China (n=29, 34.9%), France (n=10, 12.1%) and Belgium (n=10,12.1%) being the top contributing countries.

In this bibliometric analysis, we found that 54.8% of the total patients were males while 45.2% were female patients. An article written by Bwire, it was reported that females were more resistant to Covid-19 infection. This could be attributed to various factors such as sex hormones, higher expression of ACE-2 receptors in men and also difference in the lifestyles. ^7^

The incidence of COVID-19 infection was highest in adults between 26-65 years (63.2%), followed by the adults over 65 years of age (26.8%), and least in 12-25 years age group (9.8%).

Similar findings were reported in a study conducted by Jakhmola S et al, with the highest incidence of Covid-19 infection in the age groups of 20-49 years and above 50 years, and the least in the paediatric age group. This could possibly be due to lesser expression of the coronavirus (ACE-2) receptors in the nasal epithelium in younger age groups, leading to reduced susceptibility to Covid 19 infection. ^8^ Centers for Disease Control and Prevention (CDC) reported the highest incidence of COVID-19 infection among adults above the age of 80 years and in the age group of 18-24 years during 2020. ^9^

In our analysis, the most common clinical features were fever (28.2%), tachycardia (17.9%) and dry cough (13.8%). Other common features included impaired sense of smell (1.8%), impaired taste sensation (1.8%) and headache (1.5%). Similar to the above-mentioned findings, a study by Cascella M et al, reported the most common symptoms in patients to be fever, dry cough and dyspnoea. Other lesser common symptoms included malaise and headache. ^10^ In a study by Mullol J et al., it was concluded that most viral respiratory infections such as COVID-19 are associated with impairment of sense of smell. The incidence of olfactory and gustatory symptoms varies due to the varied methodology used in various studies. ^11^

Of the total patients analysed in our study, 97.8% patients were found to have comorbidities. Of which, hypertension (30.6%), diabetes mellitus (23.1%) and obesity (12.1%) were the commonest comorbidities found in the patients. Other comorbidities included chronic liver disease (0.34%), endocrine disorders (0.24%) and psychiatric disorders (0.06%). In a study by Sanyaolu et al, that the common comorbidities found were hypertension, diabetes mellitus and obesity, which were associated with poorer outcomes in COVID-19 patients. ^12^

Of all the patients analysed in our study, 13.9% of patients developed complications following COVID-19 infection. The common complications seen among the patients were acute kidney injury (22.5%), ARDS (18.3%) and sepsis (11.0%). Other complications included thrombosis (3.6%), acute hepatic injury (2.4%) and arrhythmias (2.0%). In the study by Cascella M et al, it was reported that ARDS was the most common pulmonary complication of COVID-19 while extra-pulmonary complications included AKI, cardiovascular complications and prothrombotic complications. ^10^ Similarly, in another study by Isath A et al., the most common complications reported were respiratory failure, AKI, sepsis and thrombosis. ^13^

In our analysis, 57.8% patients recovered without any complications, while 31.1% were hospitalised, and 11.1% deceased due to the illness. Similar findings were reported in a study by Isath A et al., where 51.4% patients recovered and were discharged home, and the overall inpatient mortality was reported to be 13.2%. Most of the patients with comorbidities were associated with higher number of complications and mortality. ^13^

## Conclusion

Analyzing the 50 most-cited articles in COVID-19 and the complications of COVID-19 and assessing their citations-per-year is a valuable approach for clinicians and researchers. It offers a quick overview of the most influential literature and COVID-19 research has evolved over time. Moreover, a detailed review of these articles can uncover the key attributes that make papers highly cited. This insight can be instrumental for authors, offering guidance on creating impactful research in the future. Overall, this method serves as a strategic tool for staying updated and contributing meaningfully to the field of COVID-19 research.

## Data Availability

Dryad: Data of top 50 most cited articles about COVID-19 and the complications of COVID-19, https://doi.org/10.5061/dryad.tx95x6b4m. ^14^ This project contains the following underlying data:
-
Data_of_top_50_most_cited_articles_about_COVID-19_and_the_complications_of_COVID-19.xlsx Data are available under the terms of the Creative Commons Zero “No rights reserved” data waiver (CC0 1.0 Public domain dedication).
