# Supplementary material for: The top 50 most-cited articles about COVID-19 and the complications of COVID-19: A bibliometric analysis
Source: F1000Res. 2024 May 3;13:105. Originally published 2024 Feb 16. [Version 3] doi: 10.12688/f1000research.145713.3 (PMC11325134; doi:10.12688/f1000research.145713.3)
Supplement: Peer review history

# Reviewer report: Reviewer response for version 3

**DOI:** 10.5256/f1000research.165741.r260932

**Published:** 2024-08-14

**Authors:** Chen Liu

**Affiliations:** 1 department of radiology, Third Military Medical University, Chongqing, China

The article provides a bibliometric analysis of the 50 most-cited articles on COVID-19 complications, aiming to highlight influential studies and identify factors contributing to high citation rates. The authors successfully analyze the publication trends, study characteristics, and demographic data related to these articles, offering insights into the multifaceted impacts of COVID-19 across various organ systems. The study emphasizes the significant role of publications from China and fields such as Internal Medicine and Pulmonary Medicine.

 Detailed Comments:

1. **Presentation and Literature Citation:**The work is generally well-organized, providing a comprehensive overview of the top-cited literature on COVID-19 complications. The inclusion of various complications across multiple organ systems makes the analysis robust and relevant.
2. **Study Design and Technical Soundness:** The study design is appropriate for a bibliometric analysis, and the work is technically sound. The authors have employed a rigorous selection process, which is crucial for ensuring the relevance and quality of the articles analyzed.
3. **Methodology and Replicability:**The authors describe their methodology in detail, particularly in terms of the criteria for article selection and the parameters analyzed.
4. **Source Data Availability:**The authors have provided a comprehensive dataset of the top 50 most-cited articles, which serves as a valuable resource for further research.
5. **Conclusions and Support from Results:** **Positive Aspects:** The conclusions drawn are adequately supported by the results, reflecting a clear understanding of the factors contributing to the citation impact of COVID-19 research. The discussion around the predominance of studies from certain countries and fields provides valuable insights. **Constructive Feedback:** The authors might consider expanding their conclusions to reflect on how these citation patterns could influence future research priorities and the potential biases in the current COVID-19 literature.

Is the work clearly and accurately presented and does it cite the current literature?

Yes

If applicable, is the statistical analysis and its interpretation appropriate?

Not applicable

Are all the source data underlying the results available to ensure full reproducibility?

Partly

Is the study design appropriate and is the work technically sound?

Yes

Are the conclusions drawn adequately supported by the results?

Yes

Are sufficient details of methods and analysis provided to allow replication by others?

Yes

Reviewer Expertise:

Strengths：                                                                          Comprehensive Review: The study effectively identifies and analyzes the top 50 most-cited articles on COVID-19 complications, providing valuable insights into the most influential research in this field.Methodological Rigor: The use of Scopus as a data source and the detailed criteria for article selection and evaluation ensure a high level of methodological rigor.Detailed Analysis: The study provides extensive data on various aspects of the articles, including publication year, journal impact, geographical distribution, and study focus, offering a thorough overview of the research landscape.Clinical Relevance: By focusing on complications and clinical features, the study provides information that is directly relevant to healthcare professionals dealing with COVID-19 patients.                         Recommendations：                                                           Expand the Time Frame: The study focuses on articles published up to July 2021. Extending the analysis to include more recent publications would provide a more current view of influential research.Include More Databases: Incorporating other databases like PubMed and Web of Science could offer a broader scope and capture additional influential articles not indexed in Scopus.Deeper Analysis of Complications: A more detailed analysis of specific complications and their management could enhance the practical utility of the study for clinicians.Visualization Tools: Utilizing visual tools like graphs and charts to represent data on citations, geographical distribution, and study focus could make the findings more accessible and easier to interpret.Addressing Bias: Discuss potential biases, such as the overrepresentation of certain countries or journals, and suggest ways to mitigate these in future research.         Limitations：                                                             Publication Bias: The study may be influenced by publication bias, where more prominent journals and articles with positive findings are more likely to be cited.

I confirm that I have read this submission and believe that I have an appropriate level of expertise to confirm that it is of an acceptable scientific standard.

# Reviewer report: Reviewer response for version 3

**DOI:** 10.5256/f1000research.165741.r290243

**Published:** 2024-08-14

**Authors:** Pinar Yildiz Gülhan

**Affiliations:** 1 Duzce University, Konuralp, Duzce, Turkey

The article provides a valuable overview of the 50 most cited articles on COVID-19 and its complications, highlighting key trends in publication, authorship, and research focus. The analysis of patient demographics, clinical features, and comorbidities offers insights into the factors influencing disease severity and outcomes. The discussion on complications and treatment options further enhances the clinical relevance of the study. However, the article could be strengthened by incorporating visual aids, expanding the discussion of certain findings, and providing more specific recommendations for future research. Overall, despite some limitations, the study serves as a useful resource for researchers and clinicians seeking a concise overview of the influential literature on COVID-19 complications. Tables can be visualised with VOSviewer and other bibliometric analysis programs.

Is the work clearly and accurately presented and does it cite the current literature?

Yes

If applicable, is the statistical analysis and its interpretation appropriate?

Yes

Are all the source data underlying the results available to ensure full reproducibility?

Yes

Is the study design appropriate and is the work technically sound?

Yes

Are the conclusions drawn adequately supported by the results?

Yes

Are sufficient details of methods and analysis provided to allow replication by others?

Yes

Reviewer Expertise:

ı m a pulmonogist and my research area is molecular biology

I confirm that I have read this submission and believe that I have an appropriate level of expertise to confirm that it is of an acceptable scientific standard, however I have significant reservations, as outlined above.

# Reviewer report: Reviewer response for version 3

**DOI:** 10.5256/f1000research.165741.r273085

**Published:** 2024-05-10

**Authors:** Husnul Khuluq

**Affiliations:** 1 Universitas Muhammadiyah Gombong, Kebumen, Indonesia

The third revision of the document is substantially complete; however, it lacks a crucial element in the conclusion section, specifically concerning recommendations for future research.

Is the work clearly and accurately presented and does it cite the current literature?

Partly

If applicable, is the statistical analysis and its interpretation appropriate?

I cannot comment. A qualified statistician is required.

Are all the source data underlying the results available to ensure full reproducibility?

Yes

Is the study design appropriate and is the work technically sound?

Partly

Are the conclusions drawn adequately supported by the results?

Partly

Are sufficient details of methods and analysis provided to allow replication by others?

Yes

Reviewer Expertise:

bibliometric , big data , machine learning, clinical pharmacy

I confirm that I have read this submission and believe that I have an appropriate level of expertise to confirm that it is of an acceptable scientific standard.

# Reviewer report: Reviewer response for version 2

**DOI:** 10.5256/f1000research.164836.r266882

**Published:** 2024-04-23

**Authors:** Husnul Khuluq

**Affiliations:** 1 Universitas Muhammadiyah Gombong, Kebumen, Indonesia

1. In the discussion session, the utilization of remdesivir and anticoagulants for therapy was addressed. However, this topic was not supported by bibliometric analysis data, unlike other discussed variables such as comorbidities, age, gender, clinical symptoms, complications, and outcomes. On the other hand, several other antiviral agents such as oseltamivir and favipiravir have not been discussed. Furthermore, the use of other drug classes, including corticosteroids, HCQ (Hydroxychloroquine), and antibiotics, which significantly influence therapy outcomes, was also not covered. This omission highlights a gap in the comprehensive evaluation of therapeutic strategies.

 2. In the conclusion, it would be advisable for the authors to concentrate more closely on the designated topic of the paper, which is complications. This focus would enhance the relevance and coherence of the findings in relation to the stated objectives of the research.

Is the work clearly and accurately presented and does it cite the current literature?

Partly

If applicable, is the statistical analysis and its interpretation appropriate?

I cannot comment. A qualified statistician is required.

Are all the source data underlying the results available to ensure full reproducibility?

Yes

Is the study design appropriate and is the work technically sound?

Partly

Are the conclusions drawn adequately supported by the results?

Partly

Are sufficient details of methods and analysis provided to allow replication by others?

Yes

Reviewer Expertise:

bibliometric , big data , machine learning, clinical pharmacy

I confirm that I have read this submission and believe that I have an appropriate level of expertise to confirm that it is of an acceptable scientific standard, however I have significant reservations, as outlined above.

# Response

**Published:** 2024-04-29

**Authors:** Matthew Antony Manoj

**Affiliations:** Manipal Academy of Higher Education, Manipal, Karnataka, India

We would first like to thank you for taking the time to review this version of our article. We have tried to address each comment and incorporate the same into the article. 

 Q1. In the discussion session, the utilization of remdesivir and anticoagulants for therapy was addressed. However, this topic was not supported by bibliometric analysis data, unlike other discussed variables such as comorbidities, age, gender, clinical symptoms, complications, and outcomes. On the other hand, several other antiviral agents such as oseltamivir and favipiravir have not been discussed. Furthermore, the use of other drug classes, including corticosteroids, HCQ (Hydroxychloroquine), and antibiotics, which significantly influence therapy outcomes, was also not covered. This omission highlights a gap in the comprehensive evaluation of therapeutic strategies

 A1. Thank you for the comment. In the current version of the article, we have discussed the use of anti-virals and steroids in patients with COVID-19. Additionally, we have also highlighted the current guidelines for steroid use in COVID-19 patients. 

 Q2. In the conclusion, it would be advisable for the authors to concentrate more closely on the designated topic of the paper, which is complications. This focus would enhance the relevance and coherence of the findings in relation to the stated objectives of the research.

 A2. Thank you for the comment. The conclusion has been edited to better highlight the complications of COVID-19.

# Reviewer report: Reviewer response for version 1

**DOI:** 10.5256/f1000research.159695.r253907

**Published:** 2024-04-03

**Authors:** Husnul Khuluq

**Affiliations:** 1 Universitas Muhammadiyah Gombong, Kebumen, Indonesia

The literature is up-to-date, but it is insufficient with only 14 references. Bibliometric research is a review-based study, so it requires a more extensive collection of literature than experimental or observational research.

 The study design is sound, utilizing two databases: Scopus and Web of Science. However, a drawback of using these two databases is the difficulty in visualization with tools like VOSviewer or Biblioshiny. Bibliometric research should display network visualizations among institutions, countries, and author collaborations. Utilizing only Web of Science might actually be sufficient due to its more comprehensive visualization capabilities. The main weakness of this article is the absence of such visualizations, as explained above.

 Overall, bibliometric analysis should ideally utilize applications such as VOSviewer or Biblioshiny, wherein citation counts will automatically appear. The utilization of these applications is not explicitly stated within the research methodology.

 This article bears closer resemblance to a review article rather than a bibliometric analysis.

 The literature is relevant, but the discussion lacks depth due to the scarcity of literature. The discussion on complications, which is the main topic, should be more detailed with literature from various countries, as complications may vary.

 The conclusion remains too general and is not sufficiently relevant to its title, which is complications in COVID-19 cases. Please formulate a more specific conclusion and add recommendations.

Is the work clearly and accurately presented and does it cite the current literature?

Partly

If applicable, is the statistical analysis and its interpretation appropriate?

I cannot comment. A qualified statistician is required.

Are all the source data underlying the results available to ensure full reproducibility?

Yes

Is the study design appropriate and is the work technically sound?

Partly

Are the conclusions drawn adequately supported by the results?

Partly

Are sufficient details of methods and analysis provided to allow replication by others?

Yes

Reviewer Expertise:

bibliometric , big data , machine learning, clinical pharmacy

I confirm that I have read this submission and believe that I have an appropriate level of expertise to state that I do not consider it to be of an acceptable scientific standard, for reasons outlined above.

## References

[1] : The Impact of the COVID-19 Pandemic on Cardiovascular Disease Research: A Bibliometric Analysis.225: 10.2991/978-94-6463-284-2_80 745-757 10.2991/978-94-6463-284-2_80

[2] : A bibliometric analysis of coronavirus disease (COVID-19) mortality rate. *Bali Medical Journal* .2022;11(2) : 10.15562/bmj.v11i2.3423 579-586 10.15562/bmj.v11i2.3423

# Response

**Published:** 2024-04-10

**Authors:** Matthew Antony Manoj

**Affiliations:** Manipal Academy of Higher Education, Manipal, Karnataka, India

We would like to express our gratitude to the reviewer for dedicating their time to thoroughly review our article. We have carefully examined each comment provided and have made every effort to comprehensively address each one. 

 Q. The literature is up-to-date, but it is insufficient with only 14 references. Bibliometric research is a review-based study, so it requires a more extensive collection of literature than experimental or observational research.

 Ans.

 We have now expanded our list of references and have incorporated this additional information into our discussion section to ensure a more comprehensive discussion.

 QThe study design is sound, utilizing two databases: Scopus and Web of Science. However, a drawback of using these two databases is the difficulty in visualization with tools like VOSviewer or Biblioshiny. Bibliometric research should display network visualizations among institutions, countries, and author collaborations. Utilizing only Web of Science might actually be sufficient due to its more comprehensive visualization capabilities. The main weakness of this article is the absence of such visualizations, as explained above. Overall, bibliometric analysis should ideally utilize applications such as VOSviewer or Biblioshiny, wherein citation counts will automatically appear. The utilization of these applications is not explicitly stated within the research methodology. This article bears closer resemblance to a review article rather than a bibliometric analysis.

 Ans. Thank you for your comment. We wish to clarify that we did not employ visual representation tools such as VOSviewer or Biblioshiny. The data analysis was conducted individually by each author and subsequently verified by all authors. This approach has been acknowledged as a limitation in the discussion section of our study. 

 Q. The literature is relevant, but the discussion lacks depth due to the scarcity of literature. The discussion on complications, which is the main topic, should be more detailed with literature from various countries, as complications may vary.

 Ans.

 Thank you for your comment. We've now enriched the discussion section of the article with additional information as mentioned previously. Furthermore, we wish to clarify that this bibliometric study does not focus exclusively on the complications of COVID-19. Instead, it broadly covers COVID-19 and its complications. Through our data analysis, we discovered that the complications of COVID-19 remained relatively consistent across the various countries we analyzed. Hence as a result of this, we had focussed the content in the discussion revolving around the most common types of complications in COVID-19

 Q.The conclusion remains too general and is not sufficiently relevant to its title, which is complications in COVID-19 cases. Please formulate a more specific conclusion and add recommendations.

 Ans. 

 In response to your feedback, we have now revised the conclusion to be more specific and directly relevant to the content of the article.
